# Anti-Inflammatory and Antioxidant Activity of Pollen Extract Collected by *Scaptotrigona affinis postica*: in silico, in vitro, and in vivo Studies

**DOI:** 10.3390/antiox9020103

**Published:** 2020-01-24

**Authors:** Alberto Jorge Oliveira Lopes, Cleydlenne Costa Vasconcelos, João Batista Santos Garcia, Myssa Sued Dória Pinheiro, Francisco Assis Nascimento Pereira, Darleno de Sousa Camelo, Sebastião Vieira de Morais, José Roberto Brito Freitas, Cláudia Quintino da Rocha, Maria Nilce de Sousa Ribeiro, Maria do Socorro de Sousa Cartágenes

**Affiliations:** 1 Experimental Study of Pain Laboratory, Department of Physiological Sciences, Federal University of Maranhão, São Luís 65080-805, Brazil; cleydlenne@yahoo.com.br (C.C.V.); jbgarcia@uol.com.br (J.B.S.G.); leno.camelo@gmail.com (D.d.S.C.);; 2 Laboratory of Pharmacognosy, Department of Pharmacy, Federal University of Maranhão, São Luís 65080-805, Brazil; myssa.sued@hotmail.com (M.S.D.P.);; 3 Center for Agricultural and Environmental Sciences, Campus IV, Federal University of Maranhão, Chapadinha 65500-000, MA, Brazil; 4 Chemistry Department, Federal University of Maranhão, São Luís 65080-805, Brazil

**Keywords:** stingless bee pollen, biological activity, pain, molecular docking, tubi

## Abstract

Bees are of great importance for plant diversity for being an important pollinating agents. Stingless bees such as *Scaptotrigona affinis postica*, is cultivated largely due to the products offered by it. Pollen is one of these products, which has been highlighted for exhibit various therapeutic properties. Considering the bioactivity of this natural product, this study investigated the antioxidant, anti-inflammatory, antinociceptive activities, and elucidated the chemical composition of pollen collected extract by *Scaptotrigona affinis postica*. Using in vitro assays, the antioxidant potential and inhibitory activity against the COX enzyme from pollen extract was evaluated. Additionally, tests were performed to measure the anti-inflammatory and antinociceptive activities in animal models. In our results, we found that pollen extract showed antioxidant effects and inhibitory activity against the COX enzyme. The in vivo assays showed that the extract acts on the nervous system in local and systemic levels and that the anti-inflammatory activity is due the prostanoids reducing. Chemical analyses recognize 10 molecules in the extract belonging to the polyphenol and flavonoids classes and the computational study suggests that is responsible for the observed results. Thus, it is reported for the first time the biological potential of *S. aff. postica* pollen extract and we conclude that this bee product can be considered as one source of potential new drugs.

## 1. Introduction

Bees represent a species with wide biodiversity in the world and have an important ecological role, as they are fundamental in maintaining plant diversity, maintaining an intrinsic relationship with these, being responsible for the pollination of several plant species [1]. Bee products have been widely used for generations in human health due to their recognized therapeutic and nutritional properties [2]. Bee pollen is a complex blend of flower pollen, nectar, and enzymes and other bee secretions [3]. The significant amount of compounds such as phytosterols, carbohydrates, enzymes, phenolic compounds, flavonoids, nucleic acids, triterpenes, vitamins, and other biologically active substances present in bee pollen provides several activities, including anti-inflammatory and antioxidant activity, thus demonstrating considerable biological potential which justifies their use for the research of new pharmacological resources [4].

Stingless bees are popularly known as meliponines, and belong to the Meliponinae subfamily which is divided into two tribes: Meliponini and Trigonini, with widespread occurrence in countries from South America, such Bolivia, Peru, and Brazil [5]. In Brazil, there is a great diversity of native stingless bees, and in the North and Northeast regions a large number of species are concentrated. In the state of Maranhão, the most relevant species are *Melipona fasciculata* Smith 1858 (Apiade, Meliponini) popularly known as tiúba, and *Scaptotrigona affinis postica* Latreille, 1807 (Apidae, Meloponini) popularly known as tubi [6,7].

Although *M. fasciculata* is the stingless bee where most research involving the biotechnological potential of this species is concentrated [7,8,9,10,11], other species such as *S. aff. postica* can be the focus of innovative research being an important species with potential for chemical and biological exploration.

Our group previously reported the activity of propolis hydroalcoholic extract from *S. aff. postica*, demonstrating that this product has low toxicity even at high doses [12] as well as exhibits an anti-tumor effect and reduced nitric oxide (NO) expression in animal models [13]. However, it is not known about the biological activities of pollen collected by *S. aff. postica*.

Considering the studies previously conducted by Choi [14], Maruyama et al. [15], and Lopes et al. [7] about pollen/bee pollen bioactivity and chemical composition, it can be suggested that the collected pollen by *Scaptotrigona affinis postica* may have anti-inflammatory and antioxidant potential, being as a basis for research into new drugs used to treat inflammatory disorders.

Thus, this study evaluated for the first time the anti-inflammatory and antioxidant activity from the pollen extract collected by *S. aff. postica,* identified the compounds present in the material, and correlated which compounds may be associated with bioactivity.

## 2. Materials and Methods 

### 2.1. Pollen Collection and Extract Preparation

**Figure 1:**
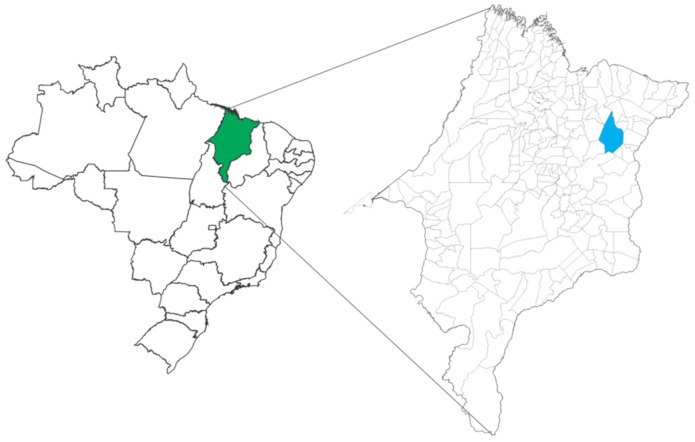
Maranhão State (in green) and the city of Chapadinha (in blue).

The pollen from *S. aff. postica* was collected using sterilized tools directly from the beehives in the stingless beehives in the city of Chapadinha (Cerrado, Brazilian savannah) (Figure 1) in the state of Maranhão, Brazil. After collection, pollen sample was identified, stored in a sterile recipient, and kept refrigerated at 4 °C until extract preparation.

The extract was obtained by pollen maceration with 70% ethanol/water (70:30, v/v) with a solid to solvent rate of 1:5 (m/v) for 3 days, with solvent renovation each 24 h. The product from all extractions was combined, filtered, and concentrated using a rotary evaporator under vacuum at 40 °C, thus obtaining the crude pollen hydroethanolic extract, which were coded for TbHPE (Tubi Hydroethanolic Pollen Extract), kept refrigerated until their lyophilization (using 15 μM of Hg at 100 °C for 48 h) and use.

### 2.2. Determination of Total Phenolic Content (TPC)

The total polyphenol content in the extract was performed using Folin–Ciocalteau reagent and 20% sodium carbonate (NaCO_3_), like was described in [7]. The TPC was calculated using a gallic acid calibration curve (2.5–40.0 μg/mL) and expressed as gallic acid equivalent (%). Analyses were performed in triplicate and the mean value was calculated for each sample [11].

### 2.3. Determinations of Total Flavonoid Concentration (TFC) 

For total flavonoid concentration we used photocolorimetric method with 5% methanolic aluminum chloride solution (AlCl_3_), like was described in [7]. The concentration was calculated from the calibration curve constructed with standard quercetin solution (Merck, Germany) (1–30.00 μg/mL) and expressed as quercetin equivalent (%). The analyses were performed in triplicate [10].

### 2.4. Determination of Antioxidant Activity

#### 2.4.1. DPPH^•^ Radical Scavenging Activity

The antioxidant activity of TbHPE was assessed using the DPPH^•^ free radical scavenging assay according to that described by [16] with modifications from [11]. The pollen extract was diluted on methanol at different concentrations ranging from 30 to 480 µg/mL and added to a methanol solution of DPPH^•^ (40.0 μg/mL) like was described in [7]. The percent inhibition was calculated using the formula

DPPH^•^ scavenging activity (%) = 100 − [(A_sample_ − A_blank_) × 100/ A_control_],

where A_sample_ = absorbance of the sample after 30 min of reaction, A_blank_ = absorbance of the blank, and A_control_ = absorbance of the control.

The percentage of scavenging activity was calculated against the sample concentration to obtain the IC_50_, defined as the concentration of sample necessary to cause 50% inhibition. All experiments were done in triplicate.

#### 2.4.2. Ferric Reducing Antioxidant Power Assay (FRAP)

FRAP measures the ferric-reducing ability of a sample in acid ambience (pH 3.6), forming an intense blue color as the ferric tripyridyltriazine (Fe^3+^-TPTZ) complex is reduced to the ferrous (Fe^2+^) form. The test was performed according Benzie et al. [17] with modifications from Dutra et al. [11]. The pollen extract was diluted like was described in [7] and the results were expressed as millimoles of Fe^2+^ per gram of sample. All experiments were done in triplicate.

#### 2.4.3. ABTS^•+^ Assay

The ABTS solution was prepared in water and potassium persulfate and kept in the dark room for 16 h before testing for the complete oxidation of ABTS^•+^ and the generation of the highly stable chromophore cation radical 2,2′-azino-bis(3-ethylbenzothiazoline-6-sulfonic acid) (ABTS^•+^) [18] with modifications [19]. The ABTS^•+^ solution was diluted with 70% ethanol/water (70:30, v/v) until the absorbance at 734 nm reached 0.7 ± 0.02, like was described in [7]. The IC_50_ values were determined for each sample, using the formula:

Scavenging ability (%) = (1 − A_sample_/A_blank_) × 100.

### 2.5. COX Inhibition Assay

The assay was performed according to the manufacturer’s recommendations (COX Colorimetric Inhibitor Screening Assay Kit—Item No. 701050-Cayman Chemical^®^), and like was described in [7], being performed in 96-well microplates and pollen extract was tested at three doses (2, 10, and 50 µg/mL) against COX-1 and COX-2.

After reagent and plate preparations following the manual of instructions, the colorimetric analysis was performed using arachidonic acid as substrate of the COX-catalyzed enzyme reaction, the plates were read at 590 nm.

### 2.6. Animals

For the in vivo assay, 60 adult male Mus musculus mice, Swiss strain, ranging from 25 to 35 g, which were procured from the Central Vivarium (Biotério Central) of Federal University of Maranhão (UFMA) were used. Animals were maintained in *n* = 5 per box and provided food and water ad libitum in an environment with 12/12 h light/dark cycle at 22 °C. All protocols used were performed according to the recommendations of IASP Guidelines for the Use of Animals in Research and with National Council for the Control of Animal Experimentation (CONCEA) and were authorized by the UFMA Ethics Committee in Animal Use (ECAN), ruling no. 64/2016, protocol no. 23115.016655/2016–83.

### 2.7. Anti-Inflammatory Activity

#### 2.7.1. Carrageenan-Induced Paw Edema Test

Mice were randomized to groups (*n* = 5) treated orally with vehicle (saline) (10 mL/kg), TbHPE (250 and 500 mg/kg) or indomethacin (10 mg/kg). Paw edema induction was undertaken by administration from 50 µL of 1% carrageenan in the subplantar region from the animal paw. This induction was performed 60 min after the administration of TbHPE, indomethacin or saline. A digital plethysmometer (Ugo Basile Model, Verese, Italy) was used to evaluate the variation of edema for 5 h, with a 60 min interval between each measurement. The difference between induced paw-volume versus basal time in each evaluation hour was used to evaluate the effect of treatment [20,21]. 

#### 2.7.2. Dextran-Induced Paw Edema Test

The test was used to evaluate pharmacological activity from subplantar administration of 1% dextran. Mice were randomized into groups (*n* = 5) treated orally with vehicle (saline) (10 mL/kg), TbHPE (250 and 500 mg/kg) or cyproheptadine (10 mg/kg). Paw edema induction was procedure by administration from 50 µL of 1% dextran in the subplantar region from the animal paw. This induction was performed 60 min after the administration of TbHPE, cyproheptadine, or saline. A digital plethysmometer (Ugo Basile Model, Verese, Italy) was used to evaluate the variation of edema for 5 h, with 60 min of interval between each measurement. The difference between induced paw-volume versus basal time in each evaluation hour was used to evaluate the effect of treatment [22].

### 2.8. Anti–Nociceptive Activity

#### 2.8.1. Acetic Acid Writhing Test

To perform this test, 1 h before writhes induction with acetic acid solution at 0.8% (10 mL/kg) by intraperitonial administration, the mice (*n* = 5) were treated orally with TbHPE 250 mg/kg, indomethacin (10 mg/kg) or vehicle (saline) (10 mL/kg). During the first 20 min after induction, the animals were evaluated regarding the number of writhes [21,23].

#### 2.8.2. Formalin Test

One hour after the oral treatment with TbHPE 250 mg/kg, indomethacin (10 mg/kg), or vehicle (saline) (10 mL/kg), the mice (*n* = 5) were administrated, by subplantar injection, 20 μL of formalin 2.5% in the right paw and the animal responses in the first 5 min (neurogenic phase) and 15–30 min after the induction (peripheral phase) were evaluated [21,24].

### 2.9. HPLC-ESI-MS/MS Analysis

The chemical analysis from TbHPE was performed by HPLC (LC-20AD, Shimadzu Corp, Kyoto, Japan) using a column Phenomenex Luna C-18 (250 × 4.6 mm, 5 µm) at 25 °C, with mobile phase ultrapure water containing 0.1% formic acid (A) and methanol (B), linear gradient applied: 0 min, 5% B; 1−60 min, 5%−100% B; 60−70 min, 100% B at flow of 1 mL/min. The liquid chromatography was coupled to a mass spectrometer (Amazon Speed ETD, Bruker, Massachusetts, USA) with electrospray ionization (ESI) and an ion-trap (IT) analyzer in negative mode, using the conditions: 4.5 kV and 325 °C capillary voltage and temperature, respectively, entrainment gas (N_2_) flow 12 L/min, nitrogen nebulizer pressure at 27 psi. The acquisition range was m/z 100–1200, with more than two events.

### 2.10. Computational Study

#### 2.10.1. Predictive Models and Theoretical Calculations

The geometric, electronic, and vibrational properties from identified compounds in TbHPE were calculated and optimized using the Gaussian 09 (Gaussian, Inc., Wallingford CT, USA) [25]. The 3D structural models were performed using GaussView 5.0.8 (Gaussian, Inc., Wallingford CT, USA) [26]. The Functional Density Theory (DFT) method, combining the functional hybrid B3LYP and the set of basis 6–31 ++ G (d, p) was used to perform the geometric optimization calculations.

#### 2.10.2. Molecular Docking

Autodock 4.2 was used to perform all molecular docking procedures [27,28]. COX-2 structure was obtained on Protein Data Bank (PDB ID 1DDX) with original ligands and artifacts removed and compound from TBHPE prepared with AutoDock Tools (Scripps Research, San Diego, California, USA) version 1.5.6 [29]. Docking protocol described in literature was used [30] with adjustment [7,21,31]. We used the oxygen atom from residue Arg120, catalytic site residue from COX-2, to position the grid box and Lamarckian genetic algorithm (LGA), with 100 runs for each compound. The criteria of lowest energy combined with visual inspection were chosen to find initial coordinates of COX-2 and TbHPE secondary metabolite interactions. 

### 2.11. Statistical Analysis

Analysis of variance (ANOVA) followed by Tukey test was used to perform the statistical analyses of the differences between experimental groups. A *p* value of <0.05 was considered as indicative of statistical significance. The data obtained were analyzed through the software GraphPad Prism 7.0^®^ (GraphPad Software, San Diego, California, USA).

## 3. Results

### 3.1. Total Phenolic Content, Total Flavonoids Content, and Antioxidant Activity

The TbHPE total phenolic content was 9.3%, total flavonoids content was 0.4%. Regarding the antioxidant activity, DPPH^•^ and ABTS^•+^ IC_50_ was 273.08 and 87.29 μg/mL, respectively, while FRAP was 0.71 mmol/Fe^2+^/g. The TbHPE and Trolox results are presented in Table 1.

### 3.2. COX-1 and 2 Inhibition Assay

The TbHPE showed inhibitory activity against both COX isoforms (COX-1 and COX-2), the high inhibition being detected with 50 µg/mL doses showing 96% and 86% inhibition from COX-2 and COX-1, respectively. At other concentrations, the inhibitory activity does not show expressive results (Table 2).

Due to the encouraging results of total polyphenol and flavonoid contents, antioxidant activity and inhibitory potential against COX enzymes, we conducted an in vivo study to evaluate the anti-inflammatory potential of TbHPE in animal models.

### 3.3. In Vivo Anti-Inflammatory Activity

#### 3.3.1. Carrageenan-Induced Paw Edema Test

**Figure 2:**
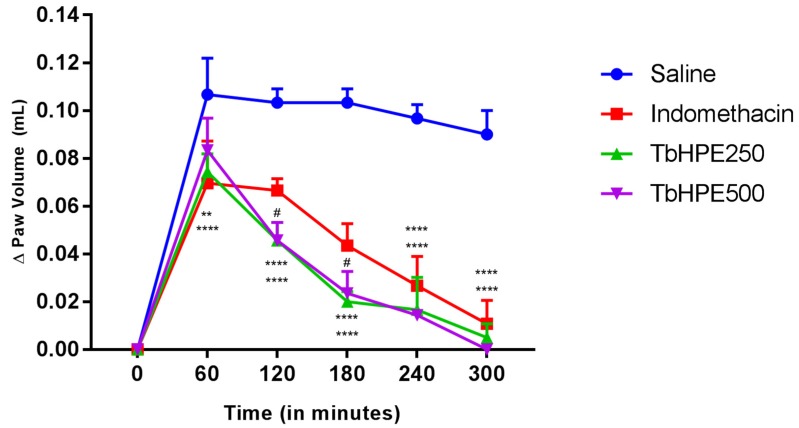
Effects of oral treatment with hydroethanolic pollen extract collected by *Scaptotrigona affinis postica* (TbHPE) against carrageenan-induced paw edema. Mice treated with saline, indomethacin (10 mg/kg), TbHPE 250 and 500 mg/kg. ** *p* < 0.01; **** *p* < 0.0001 vs. saline; # *p* < 0.05; vs. indomethacin (ANOVA; Tukey).

Treatment with TbHPE in both doses showed statistical difference for the saline group in all assessed times (*p* < 0.0001), reducing the edema in 36%, 54%, 76%, 81%, and 100% compared to saline in the 1, 2, 3, 4, and 5 hours respectively. TbHPE 250 and 500 mg/kg also shows statistical difference to indomethacin, reducing the edema in 41% and 37% in 2 and 3 h after treatment, respectively (*p* < 0.05). In the 4 and 5 h, the TbHPE provides statistically similar effects with indomethacin. In all times, there was no evidenced statistical differences between 250 and 500 mg/kg from TbHPE. These results are shown on Figure 2. 

#### 3.3.2. Dextran-Induced Paw Edema Test

**Figure 3:**
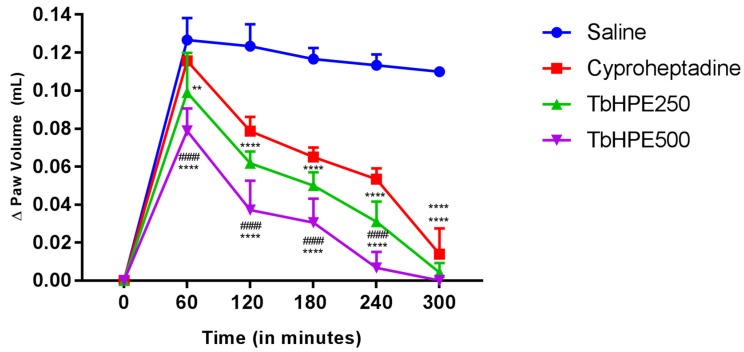
Effects of oral treatment with hydroethanolic pollen extract collected by *Scaptotrigona affinis postica* (TbHPE) against dextran-induced paw edema. Mice treated with saline, cyproheptadine (10 mg/kg), TbHPE 250 and 500 mg/kg. ** *p* < 0.01; **** *p* < 0.0001 vs. saline; #### *p* < 0.0001; vs. cyproheptadine (ANOVA; Tukey).

In all assessed times, TbHPE treatment at both doses was statistically different from the saline (Figure 3). Compared to the saline group, TbHPE 250 mg/kg produced a reduction in paw edema that varied between 30% and 100% from 1–5 h, while the TbHPE 500 mg/kg at the same time reduces the edema between 41% and 100%, also compared to the saline group. Treatment with TbHPE 250 mg/kg was efficient in reducing edema being statistically equal to cyproheptadine at all evaluated moments, while the TbHPE 500 mg/kg in 1–4 h after induction was significantly better to cyproheptadine (Figure 3). Thus, we found that TbHPE reduced the paw edema caused by subplantar administration of dextran at 1% more efficiently than cyproheptadine, showing statistically different anti-inflammatory activity than saline and cyproheptadine within the first hour after induction. Cyproheptadine only showed statistical differences compared to the saline group after the second hour of test.

### 3.4. In Vivo Anti-Nociceptive Activity

#### 3.4.1. Acetic Acid Writhing Test

**Figure 4:**
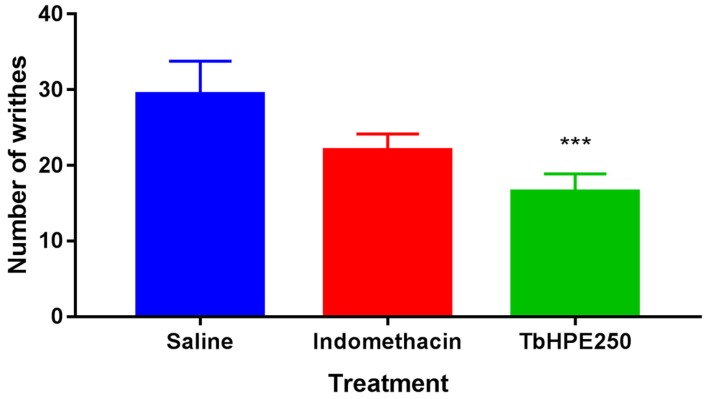
Effects of oral treatment with hydroethanolic pollen extract collected by *Scaptotrigona affinis postica* (TbHPE) on control of writhing induced by administration of 0.8% acetic acid. Mice treated with saline, indomethacin (10 mg/kg), TbHPE 250 mg/kg. *** *p* < 0.001 vs. saline; (ANOVA; Tukey).

The use of TbHPE 250 mg/kg reduces the number of abdominal writhing’s by 52% compared with the saline group (*p* < 0.001). In addition, treatment was statistically equal to indomethacin (Figure 4).

#### 3.4.2. Formalin Test

**Figure 5:**
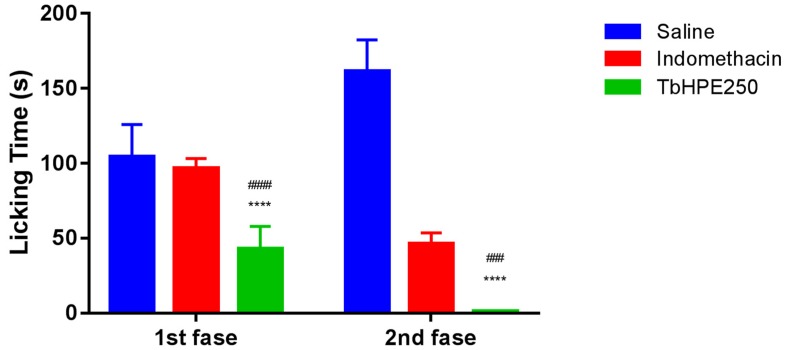
Effects of oral treatment with hydroethanolic pollen extract collected by *Scaptotrigona affinis postica* (TbHPE) on **the** formalin test induced administration of 2.5% formalin. Mice treated with saline, indomethacin (10 mg/kg), and TbHPE 250 mg/kg. **** *p* < 0.001 vs. saline; ### *p* < 0.005; #### *p* < 0.001 vs. indomethacin (ANOVA; Tukey).

The TbHPE 250 mg/kg treatment was effective in reducing licking/biting responses in both phases of the formalin-induced nociception test. In the initial phase of test (neurogenic, 0–5 min) TbHPE decreased pain response time by 57% compared to the saline group (*p* < 0.001) and by 51% compared to the indomethacin group (*p* < 0.001). In the inflammatory phase (15–30 min) from formalin test, the TbHPE 250 mg/kg decreased by 99% the response compared to the saline group (*p* < 0.001) and decreased by 96% the paw licking/biting compared to the indomethacin group (*p* < 0.005). This result indicates that TbHPE has neurogenic and peripheral effects (Figure 5).

### 3.5. LC-ESI-IT-MS/MS Analysis

**Figure 6:**
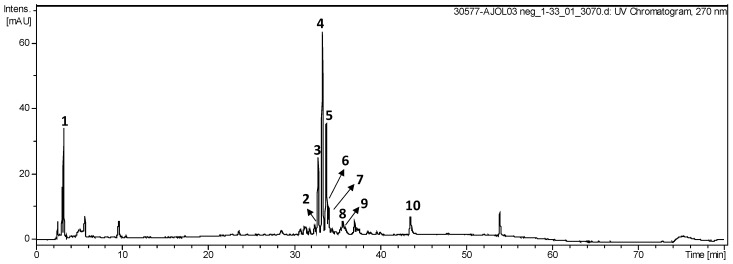
HPLC chromatograms of phenolic compounds detected at 270 nm in the hydroethanolic pollen extract collected by *S. aff. postica.*

**Figure 7:**
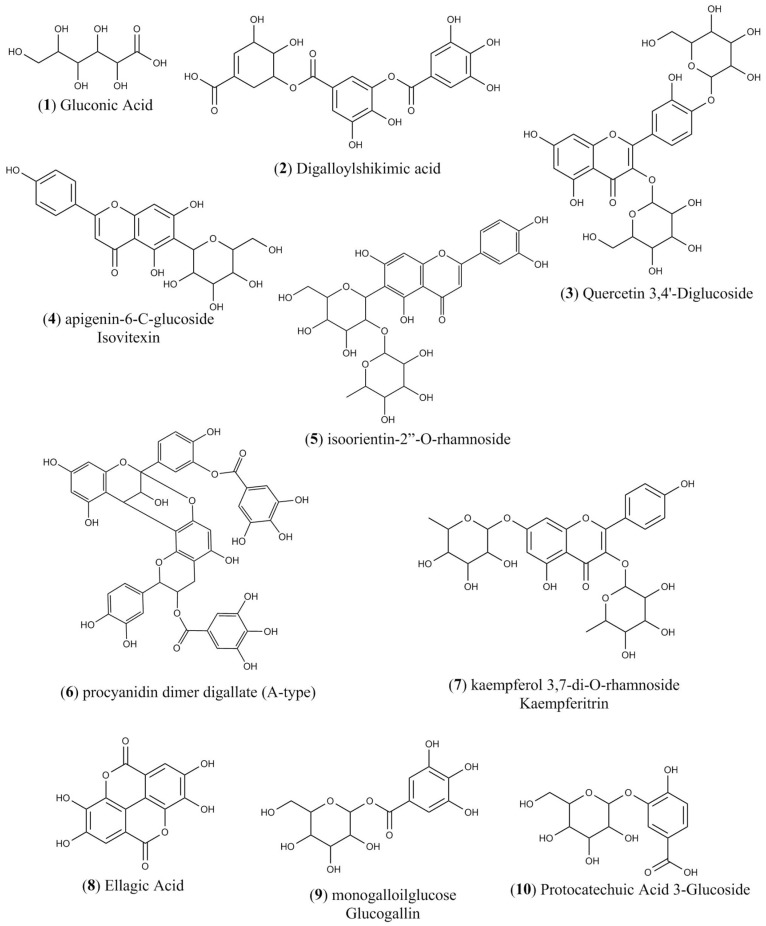
Chemical structures of the compounds identified by LC-ESI-IT-MS/MS in the hydroethanolic pollen extract collected by *S. aff. postica* from Chapadinha-MA.

Figure 6 shows the HPLC chromatogram of phenolic compounds present in TbHPE. Table 3 compiles the identified phenolic compounds, their retention time, molecular weight, molecular ion [M − H] −, and main product ions obtained by LC-MS/MS for the 10 fragmentation peaks of TbHPE. The compounds were tentatively identified by comparing their fragmentation profiles with the compounds described in literature data (Figure 7).

### 3.6. Molecular Docking

**Figure 8:**
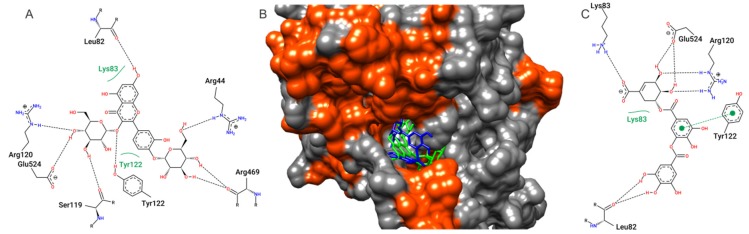
Two-dimensional diagram from contacts of COX-2 active site amino acids residues with quercetin 3,4’-diglucoside (**A**) and digalloylshikimic acid (**C**). Dashed black line—hydrogen bonds; full green lines—van der Waals interactions. In (**B**), the surface representation of quercetin 3,4’-diglucoside (green) and digalloylshikimic acid (blue) docking positions on COX-2 active site are shown.

In the molecular docking (MD) study, all compounds identified by HPLC-MS/MS on TbHPE were used. All compounds showed highest affinity parameters with the COX-2 structure, quercetin 3,4’-diglucoside and digalloylshikimic acid being the molecules that exhibit the most favorable interactions, with free binding energy values of −8.09 and −7.70 kcal/mol and 1.15 and 2.28 µM inhibition constant, respectively (Table 4). Other compounds present in TbHPE also presented interesting COX-2 affinity parameters, these values being very close suggesting that the anti-inflammatory activity of extract is probably mediated by the various extract compounds. In addition, molecular docking of commercial NSAID indomethacin was also performed. The results of indomethacin were close to TbHPE metabolites. The interactions performed by quercetin 3,4’-diglucoside and digalloylshikimic acid with the amino acid residues of the COX-2 active site, like Arg120 and Glu524 are shown in Figure 8.

## 4. Discussion

In the literature, we did not identify any papers reporting about the content of polyphenols and flavonoids contained in *S. aff. postica* bee pollen. The average polyphenol content for the hydroethanolic extract obtained in this work was 9.3%, while the flavonoid content was 0.4%. The average content of polyphenols and flavonoids in *S. aff. postica* propolis was reported as 11.95% and 0.55%, respectively [32]. The flavonoid content in another *S. aff. postica* propolis has also been reported ranging from 0.37% to 0.65% [33]. Although they are different samples, the results are similar to the present one. Lopes et al. [7] evaluated *Melipona fasciculata* Smith pollen extracts from different locations in Maranhão state and found a variation between 6.10% and 11.9% for total polyphenols and from 0.30% to 2.09% for flavonoids. Pollen extract from *Appis mellifera* present content phenolic compounds which amount to 1.6% on average [34]. The result found in TbHPE for both classes of compounds showed very close results and is remarkable when compared to results found by these authors.

The antioxidant activity of hydroethanolic pollen extract of *S. aff. postica* was evaluated by three methods: DPPH^•^, ABTS^•+^, and FRAP. Antioxidant activity by the DPPH^•^ method presented noteworthy results when compared to that found in *Appis mellifera* bee pollen results. The DPPH^•^ IC_50_ obtained in this study was 273.08 µg/mL, whereas in *A. mellifera* extracts there was a range from 810 to 4690 µg/mL [35]. Another study by Villarreal [36] showed that the ethanolic pollen extract of *Melipona seminigra* presented DPPH^•^ IC_50_ of 322.2 µg/mL. Our DPPH^•^ results are also more encouraging than that reported from *A. mellifera* pollen from Portugal and Turkey [37,38]. The DPPH^•^ IC_50_ of *S. aff. postica* from Chapadinha was more than 2-times that of *M. fasciculata* from the same city, suggesting that although these bees live in same environment, their habits and plants visited are distinct, which influences the chemical composition of their pollen, and justifies this difference in bioactivity. Regarding the FRAP and ABTS^•+^ assays, the results are similar to those reported for other bee species, including *M. fasciculata* [7,39,40]. Neutralization of free radicals is important, since these radicals promote molecular disorders that can cause various diseases [41]. According to Soares [42] free radicals are characterized as ions or atoms that have one or more unpaired electrons in the outer orbital and are very reactive. On the other hand, a substance with antioxidant capacity is one that even in a small concentration when compared to the oxidizable substrate, minimizes or extinguishes oxidative damage. 

The ability of these natural extracts to interact with free radicals involved in establishment and maintenance of inflammatory conditions, encourage studies aimed at evaluating the anti-inflammatory activity of these extracts, such as inhibition of formation from inflammatory mediators derived from COX/LOX metabolism.

Due to perform metabolism of arachidonic acid and production of prostaglandins, prostacyclins, and thromboxanes, COX is an essential component of inflammatory process and is therefore the target of inhibition of NSAIDS. However, this enzyme is also associated with other physiological processes besides the pathological process of inflammation. In present study, TbHPE when using in vitro assessment against COX inhibition produced at 50 µg/mL an inhibition rate of 86% on COX-1 and 91% against COX-2. These results are different from those obtained with *M. fasciculata* pollen collected in same city [7] with TbHPE, which showed affinity for COX-2, inhibiting 100% of this enzyme at 10 µg/mL and only 35% of COX-1. They also differ from the pollen recorded by *Cistus* sp. which showed affinity for COX-2 inhibition [15]. The suppression of the COX gene produced by pollen extract is suggested to cause the anti-inflammatory effect of this product [43]. These findings validate the hypothesis that the TbHPE has anti-inflammatory activity. 

The carrageenan-induced paw edema test is a traditional protocol for anti-inflammatory activity assessment that has two inflammation stages, the first occurring 1 h after induction, and the edema is characterized mainly by the release of vasoactive amines (histamine and serotonin) and kinins, and the second phase, from 3 h after edema induction, it is characterized by increased activity of COX-2, which will produce large amounts of prostaglandins, as well the release of nitric oxide [44,45]. There are no reports in the literature regarding the anti-inflammatory activity in vivo of TbHPE, however, it is known that the propolis extract from *S. aff. postica* decreased oxide nitric production by macrophages in mice, suggesting a possible anti-inflammatory activity [12]. TbHPE show statistical differences in reducing edema compared to saline and indomethacin treatments 2 h after induction, confirming that the extract acts by COX-2 inhibition and suggests that such propolis extract from *S. aff. postica*, pollen also probably acts by NO release inhibition, and inhibits the release of histamine and serotonin. The pollen from *M. fasciculata* also show similar results in this same test [7] and the result of TbHPE 250 mg/kg was more efficient than reported for *Cistus* sp pollen [15].

On dextran-induced paw edema test, TbHPE 250 and 500 mg/kg decreased edema from 1 to 5 h after induction, TbHPE 500 mg/kg being statically more effective that cyproheptadine from 1 h. Unlike carrageenan-induced paw edema, dextran-induced paw edema does not have two distinct phases, and this model is characterized by vasodilatation and increased vascular permeability, promoted by the release of vasoactive amines (histamine and serotonin) from mast cell degranulation, not presenting the phase normally associated with the production of prostaglandins and leukocyte infiltration [22]. According to our results, we can evidence that TbHPE also acts by release inhibition of histamine and serotonin. Pollen extract from *M. fasciculata* also show high anti-inflammatory activity in the same model [7].

The acetic acid-induced abdominal writhing test [23] is a commonly used test to evaluate analgesic activity of drug candidates on sensitization of peripheral nociceptors. Acetic acid pain occurs due to the establishment of an acute inflammatory reaction triggered by increased release of inflammatory mediators such as prostaglandins, resulting from increased COX-2 activity [46,47]. TbHPE 250 mg/kg showed a decrease in the writhing count compared to saline group and was statistically equal to indomethacin, suggesting that TbHPE contributed to palliative control of mild or moderate pain caused by phlogistic agent due to COX-2 inhibition.

The hydroethanolic pollen extract from *M. fasciculata* at 250 mg/kg reported a 54% reduction in the number of abdominal writhing compared to the saline group [7]. The result of the present study is equivalent to *M. fasciculata* pollen activity.

The first phase of formalin test occurs between 0 and 5 min after formalin injection, it is called the neurogenic phase, and occurs due to direct chemical stimulation of afferent C-type nociceptors and substances that produce local responses. The second phase occurs between 15 and 30 min after induction and represents a type of inflammatory pain involving spinal cord-reinforced synaptic transmission, as well as the release of local inflammatory mediators such as prostaglandins, serotonin, histamine, and bradykinin [24]. TbHPE showed statistically significant reductions in paw licking times when compared to saline and indomethacin groups, in both times. Like TbHPE, *Pinus* spp. pollen ethanolic extract [14] and *M. fasciculata* pollen hydroethanolic extract [7] also produced a significant inhibition of both phases of formalin pain test in mice, suggesting that pollen extracts have neurogenic and peripheral effects.

The compounds identified by chemical analysis include metabolites belonging to flavonoid and polyphenol class. Besides the compounds founded on TbHPE, quercetin 3,4’-diglucoside, ellagic acid, protocatechuic acid 3-glucoside, and gluconic acid also were reported on hydroethanolic pollen extract from *M. fasciculata* from Chapadinha [7]. TbHPE metabolites have reports in the literature about antioxidant and anti-inflammatory activities. In our molecular docking results, we evidence that quercetin 3,4’-diglucoside, digalloylshikimic acid, ellagic acid, kaempferol 3,7-di-*O*-rhamnoside (kaempferitrin), apigenin-6-*C*-glucoside (isovitexin), and isoorientin-2”-*O*-rhamnoside show favorable parameters of electronic affinity with the COX-2 structure very close to each other, suggesting that anti-inflammatory activity from TbHPE may be due to various metabolites. Quercetin-3,4-diglucoside is a quercetin glycoside flavonoid, and research conducted on the antioxidant activity of compounds present in onions has shown that this compound has obtained significant results in terms of free radical scavenging [48]. Studies by Lopes et al. [7] emphasize a possible anti-inflammatory activity that this compound also presents, since among the compounds present in the extracts analyzed by this author, quercetin-3,4-diglucoside was one that presented the best interaction with COX-2 and report the variety of activities from this compound. No papers reporting about biological activities from digalloylshikimic acid were found in the literature. Thus, the present paper reports the first suggestion of biological activity for this compound. Ellagic acid, kaempferol 3,7-di-*O*-rhamnoside (kaempferitrin), apigenin-6-*C*-glucoside (isovitexin) were reported with antioxidant and/or anti-inflammatory activities by several studies [49,50,51,52,53].

Based on present data, we reinforce the hypothesis that TbHPE and its secondary metabolites have high potential to be a target of research for new drugs with antioxidant, anti-inflammatory, and antinociceptive activity.

## 5. Conclusions

The TbHPE showed high polyphenols content and antioxidant activity in all protocols evaluated. The extract also showed in vitro COX inhibition and presented anti-inflammatory and antinociceptive activity in all evaluated in vivo models. Chemical composition of extract allowed correlating which of these compounds can be correlated with these activities. Thus, it is concluded that pollen collected by *S. aff. postica* can be considered for future research using this product as new alternatives for the treatment of inflammatory diseases and it also reinforces the urgent requirement for conservation of bees in their natural environments.

## Figures and Tables

**Table 1:** Quantifications of polyphenols (TPC) and flavonoids (TFC) contents, antioxidant activity (DPPH^•^, FRAP and ABTS^∙+^ methods) from hydroethanolic pollen extract collected by *Scaptotrigona affinis postica* (TbHPE).

Sample	TPC (%) ^*^	TFC (%) ^**^	DPPH^•^IC_50_ (μg/mL)	FRAP (mmol Fe^2+^/g)	ABTS^•+^ IC_50_ (μg/mL)
TbHPE	9.30 ± 0.56	0.40 ± 0.04	273.08 ± 1.43 ^b^	0.71 ± 0.04 ^b^	87.29 ± 0.06 ^b^
Trolox	-	-	3.08 ± 0.57 ^a^	8.87 ± 0.35 ^a^	3.67 ± 0.78 ^a^

Values expressed by the mean of triplicate measurements ± standard deviation. When in same column, different letters (^a^ and ^b^) indicates a significant difference (Tukey, *p* < 0.05). (*) expressed as gallic acid equivalent; (**) expressed as quercetin equivalent. DPPH^•^, 2,2-diphenyl-1-picrylhydrazyl radical; FRAP, ferric reducing antioxidant power; ABTS^•+^, 2,2′-azinobis-3-ethylbenzotiazoline-6-sulfonic acid. (-) unrealized.

**Table 2:** COX-1 and COX-2 inhibition from TbHPE.

Isoform	% Inhibition		
	2 µg/mL	10 µg/mL	50 µg/mL
COX-1	22%	42%	86%
COX-2	0%	29%	91%

**Table 3:** Compounds identified by LC-ESI-IT-MS/MS on negative mode, in pollen extract collected by *S. aff. postica.*

Nº	Retention Time (min)	[M−H]^−^	MS^n^ Ion m/z (−)	Tentative Identification
**1**	3.0	195	285; 177	gluconic acid
**2**	32.9	477	325; 315	digalloylshikimic acid
**3**	33.1	625	301	quercetin-3,4-diglucoside
**4**	33.4	431	311	apigenin-6-*C*-glucoside
**5**	33.6	593	429; 357	isoorientin-2”-*O*-rhamnoside
**6**	33.7	879	439; 289	procyanidin dimmer digallate (a-type)
**7**	33.8	577	285	kaempferol 3,7-di-*O*-rhamnoside
**8**	35.4	301	-	ellagic acid
**9**	35.3	331	315; 271; 209	monogalloylglucose
**10**	39.7	315	299; 153	protocatechuic acid 3-glucoside

**Table 4:** Free-binding energies and inhibition constant obtained by molecular docking of the compounds identified in TbHPE with COX-2 structure.

COX-2		
Ligand	ΔGbind (kcal/mol)	Ki (μM)
quercetin 3,4’-diglucoside	−8.09	1.15
digalloylshikimic acid	−7.70	2.28
ellagic acid	−7.63	2.65
kaempferol 3,7-di-*O*-rhamnoside (kaempferitrin)	−7.59	2.75
apigenin-6-*C*-glucoside (isovitexin)	−7.54	2.99
isoorientin-2”-*O*-rhamnoside	−7.52	3.08
monogalloylglucose	−7.15	5.74
protocatechuic acid 3-glucoside	−6.85	8.65
gluconic acid	−4.48	493.23
indomethacin	−8.10	0.92
